# Intrasexual selection drives sensitivity to pitch, formants and duration in the competitive calls of fallow bucks

**DOI:** 10.1186/s12862-015-0429-7

**Published:** 2015-08-17

**Authors:** Benjamin J Pitcher, Elodie F Briefer, Alan G McElligott

**Affiliations:** Biological and Experimental Psychology, School of Biological and Chemical Sciences, Queen Mary University of London, Mile End Road, London, E1 4NS UK; Institute of Agricultural Sciences, ETH Zürich, Universitätstrasse 2, CH-8092 Zurich, Switzerland; Present address: Department of Biological Sciences, Faculty of Science and Engineering, Macquarie University, Sydney, NSW 2109 Australia

**Keywords:** Body size, Condition, Dominance, Fundamental frequency, Male–male competition, Sexual selection, Signalling, Vocal

## Abstract

**Background:**

Mammal vocal parameters such as fundamental frequency (or pitch; *f*_*o*_) and formant dispersion often provide information about quality traits of the producer (e.g. dominance and body size), suggesting that they are sexually selected. However, little experimental evidence exists demonstrating the importance of these cues in intrasexual competition, particularly *f*_*o*_. Male Fallow deer (bucks) produce an extremely low pitched groan. Bucks have a descended larynx and generate *f*_*o*_ well below what is expected for animals of their size. Groan parameters are linked to caller dominance, body size and condition, suggesting that groans are the product of sexual selection. Using a playback experiment, we presented bucks with groans that had been manipulated to alter vocal cues to these male characteristics and compared the response to the same, non-modified (natural) groans.

**Results:**

We experimentally examined the ability of bucks to utilise putative cues to dominance (*f*_*o*_), body size (formant frequencies) and condition (groan duration), when assessing competitors. We found that bucks treated groans with lowered *f*_*o*_ (more dominant), and lowered formant frequencies (larger caller) as more threatening. By contrast, groans with raised formant frequencies (smaller caller), and shorter durations (more fatigued caller) were treated as less threatening.

**Conclusions:**

Our results indicate that intrasexual selection is driving groans to concurrently convey caller dominance, body size and condition. They represent the first experimental demonstration of the importance of *f*_*o*_ in male competition in non-human mammals, and show that bucks have advanced perception abilities that allow them to extract information based on relatively small changes in key parameters.

**Electronic supplementary material:**

The online version of this article (doi:10.1186/s12862-015-0429-7) contains supplementary material, which is available to authorized users.

## Background

Acoustic signals are a vital component of intrasexual and intersexual interactions between conspecifics [1, 2]. Quality cues in signals are typically the result of physical and physiological constraints on sound production [2]. Signals of quality provide information about the sender’s phenotype and genotype [3]. Acoustic signals may encode a variety of quality indicators, including body size, dominance and fatigue [2, 4–6]. The evolution of vocalisations involves a feedback loop between production mechanisms, the acoustic structure of signals and the behaviour of receivers in response [2, 7]. Thus, the behavioural responses of receivers, based on the acoustic information in vocalisations, drives selection at the level of signal production [2]. Understanding the perception and response to acoustic signals is therefore critical to the understanding of the evolution of acoustic communication systems.

Among mammals, vocal signals serve in both mate attraction and intrasexual competition. In humans, the fundamental frequency or pitch (*f*_*o*_) of male voices negatively correlates with levels of circulating testosterone [8–11], while taller men typically speak with lower formant dispersion [10, 12–15]. Playback studies have shown that women perceive male voices with lower *f*_*o*_ and formant dispersion as being more masculine [10, 16–18]. The relationship between perceived masculinity and circulating testosterone is largely mediated by *f*_*o*_, while the relationship between speaker height and perceived masculinity is partially mediated by both formant dispersion and *f*_*o*_ [10]. Similarly, males perceive other men with voices characterised by lower *f*_*o*_ as being more physically and socially dominant [19, 20]. Males modulate their *f*_*o*_ when speaking to a competitor, lowering their *f*_*o*_ when they perceive themselves as more dominant and raising it when they perceive themselves as less dominant [19]. Indexical cues of fitness-related parameters have also been observed in the vocal variation of non-human mammals, suggesting that they play a role in mate attraction and/or intrasexual competition. Correlations between formant frequencies (and formant dispersion), with age, body size, dominance and/or reproductive success are found in a number of groups, including ungulates [4, 5, 21], carnivores [22–24], marsupials [25] and primates [26]. *f*_*o*_ may be a reliable cue to age, decreasing with age in baboons (*Papio cynocephalus ursinus*) [27] and red deer (*Cervus elaphus*) [21], while increasing with age in fallow deer (*Dama dama*) [4]. Furthermore, *f*_*o*_ is a good indicator of dominance in fallow bucks [4, 5] and crested macaques (*Macaca nigra*) [28]. However, despite this correlational evidence, to our knowledge, no study has successfully demonstrated the importance of *f*_*o*_ in male intrasexual competition in a non-human mammal using playback experiments.

Fallow deer are a highly polygynous and size-dimorphic species [29, 30]. Males (bucks) remain silent for most of the year, but vocalise (groan) intensely during the breeding season (rut) [31]. The groan comprises a series of glottal pulses, which give rise to a very low *f*_*o*_ (28.2 ± 0.3 Hz) [32]. The broadband energy of these pulses is filtered by the vocal tract, resulting in spectral peaks called formants [33]. The larynx is mobile and is retracted during vocalisations, increasing the vocal tract length by an average of 52 % [33]. This significantly lowers formant frequencies and dispersion, and potentially exaggerates the perceived body size of callers [33]. *f*_*o*_ is not correlated with body size in fallow deer [5] and furthermore, when compared to other species, the *f*_*o*_ of fallow buck groans is well below what would be expected for an animal of that size [34].

Analyses of the vocal behaviour and acoustic parameters of fallow buck vocalisations show that these vocal signals are sexually selected and play a crucial role in reproductive contexts [4, 5, 31, 33, 35]. More dominant fallow bucks have greater mating success, and higher rank is correlated to lower *f*_*o*_ in groans and, to a lesser extent, to lower formant frequencies [4, 5]. Higher ranked males commence groaning several weeks before mating starts, and long-term investment in groaning throughout the rut is related to their mating success [31]. Additionally, fallow buck groans are dynamic signals. Bucks modulate their groaning rate in relation to the presence and composition of surrounding conspecifics [35, 36], and treat higher groaning rates as more threatening [37]. Further, variation in groan quality throughout the rut appears to provide salient information about the condition of callers [37, 38]. The *f*_*o*_ of groans is typically lowest around the peak of mating activity in the middle of the rut and increases towards the end of the rut. Similarly, the number of pulses and duration of groans decreases towards the end of the rut. This decline in groan quality is likely to be indicative of the broader decline in body condition of bucks as the rut progresses [38, 39] and is perceptible to other bucks, which treat late-rut groans as less threatening than early-rut groans [37]. While groans are likely to signal information to both male and female conspecifics [35, 36], the specific parameters (apart from groaning rate [37]; e.g. *f*_*o*_ or formants) attended to by males and females, and hence the selection pressures driving the evolution of this vocal signal remain unknown.

Because fallow buck groans appear to be the product of strong sexual selection for low *f*_*o*_, low formant dispersion, long duration and high groaning rate [4, 5, 31, 33, 35, 37, 38], we experimentally investigated whether bucks use acoustic cues to dominance, size and condition to assess competitors. Using playback experiments, we previously showed that bucks attend to changes in conspecific call rate and overall call structure, thus gaining information on the motivation and condition (fatigue) of competitors [37]. Here, we tested if bucks also attend to changes in *f*_*o*_, formants and duration. We hypothesized that males would perceive conspecific groans with lower *f*_*o*_, lower formants, or longer duration, which are indicative of more dominant, larger and/or better condition animals as more threatening, compared to groans indicating less dominant, smaller and/or poorer condition bucks [4, 5, 33, 37, 38]. Accordingly, we expected bucks to display greater attention and be more likely to retreat when presented with groans that they perceive as more threatening. We predicted that bucks would be responsive to changes in *f*_*o*_, formant dispersion and groan duration, and therefore that the responses of receivers to the acoustic information are driving selection for the production of signals that effectively transmit information about caller quality and condition.

## Results

Playbacks of fallow buck groans were presented to 10 bucks during the rut. Each buck was presented with a unique set of 5 playback stimuli consisting of the following: 1) natural groans (no manipulation); 2) same groans with fundamental frequency shifted down; 3) formant frequencies shifted up; 4) formant frequencies shifted down; 5) and shortened duration. The responses measured were the latency from the beginning of the playback presentation until the subject looked towards the speaker, the duration of looking during the presentation period, and the time taken for the subject to start moving after the beginning of the playback. For each buck, the response to modified groans was compared to the response to natural groans.

### Response to playbacks of groans with lowered fundamental frequency (f_o_)

**Fig. 1:**
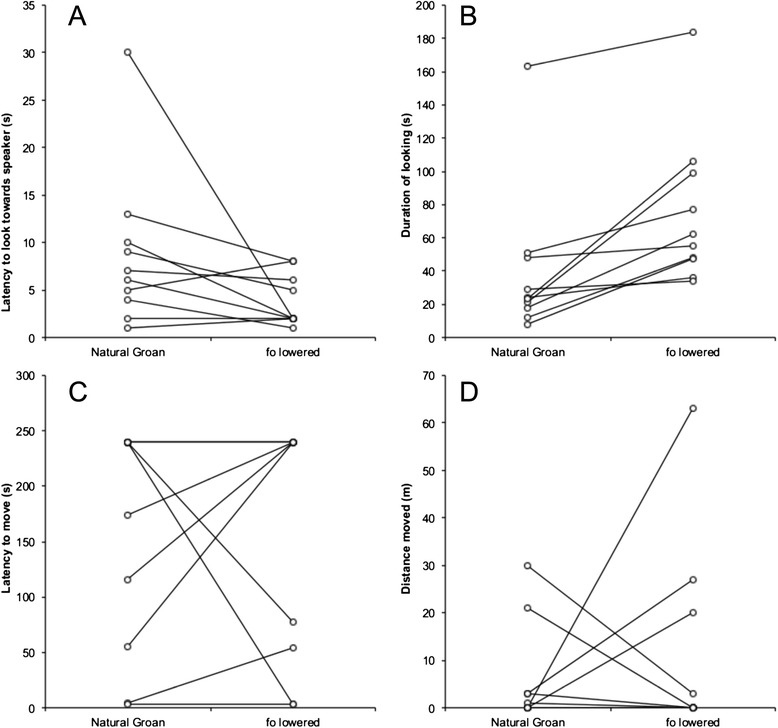
The responses of fallow bucks to natural groans and modified groans with lowered *f*_*o*_. Pairs of points joined by lines represent the response of an individual buck. Bucks responded to by looking toward the speaker faster (**a**) and for longer (**b**) in response to *f*_*o*_ lowered groans than to natural groans. There was no consistent difference in response in either the latency to move (**c**), or the distance moved (**d**) between groan types (see Results for statistics)

In response to playbacks of groans with a lower *f*_*o*_, bucks were quicker to look towards the speaker (Fig. 1a. Wilcoxon signed-ranks test: Z = -2.08, N = 10, R = 0.67, P = 0.038) and looked for longer (Fig. 1b. Wilcoxon signed-ranks test: Z = -2.80, N = 10, R = 0.89, P = 0.005), compared to natural groans. However, there was no difference between these two stimuli in the latency to move or the distance moved (latency to move: Fig. 1c. Wilcoxon signed-ranks test: Z = -0.11, N = 10, R = 0.03, P = 0.917; distance moved: Fig. 1d. Wilcoxon signed-ranks test: Z = -0.17, N = 10, R = 0.05, P = 0.866).

### Response to playback of groans with modified formant frequencies

**Fig. 2:**
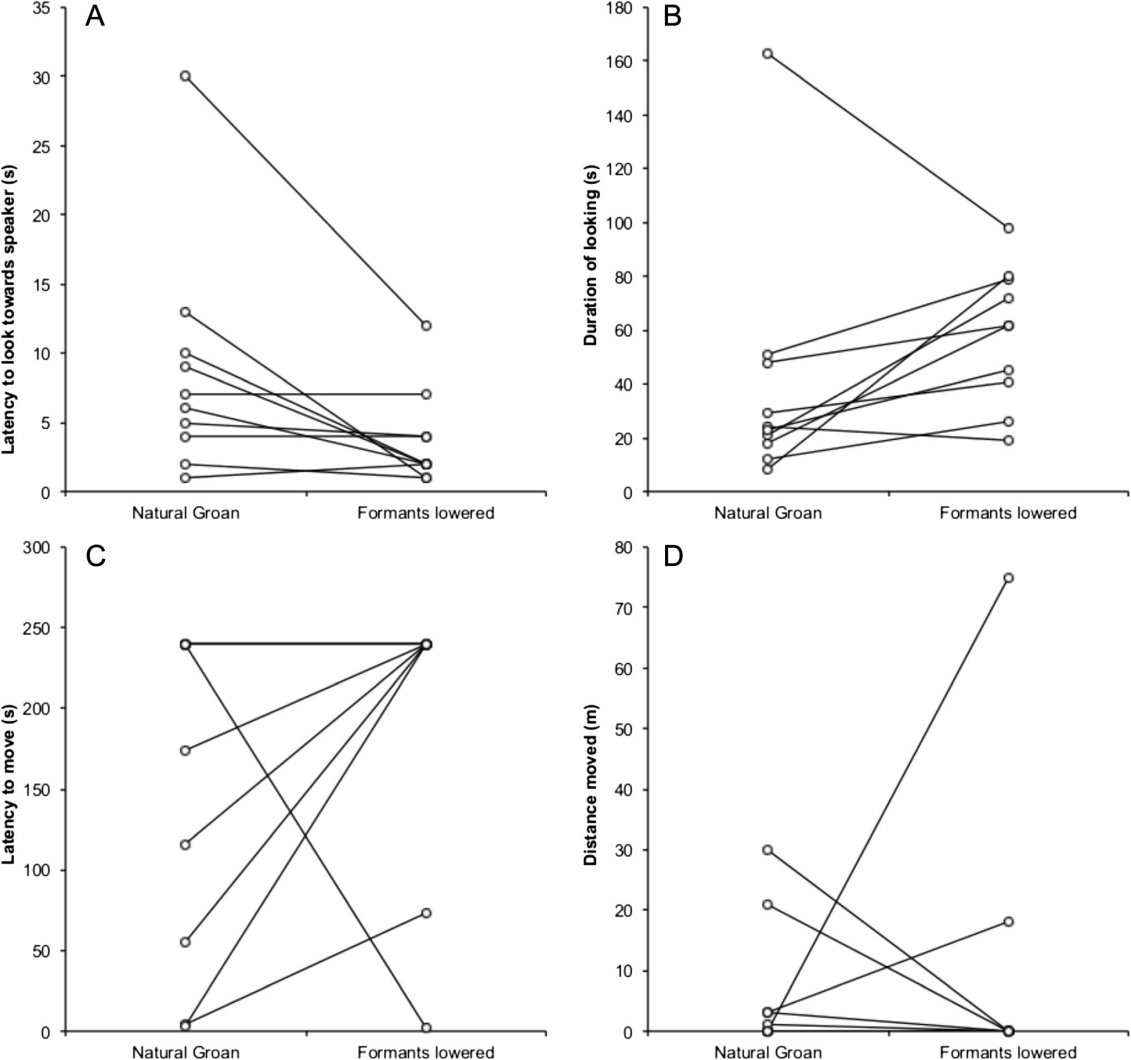
The responses of fallow bucks to natural groans and modified groans with lowered formant frequencies. Pairs of points joined by lines represent the response of an individual buck. Bucks responded to by looking toward the speaker faster (**a**) in response to lowered formant frequency groans than to natural groans. There was no consistent difference in response in the latency to move (**c**), or the distance moved (**d**) between groan types. The difference in response to the duration of looking towards the speaker (**b**) was not significant (see Results for statistics)

**Fig. 3:**
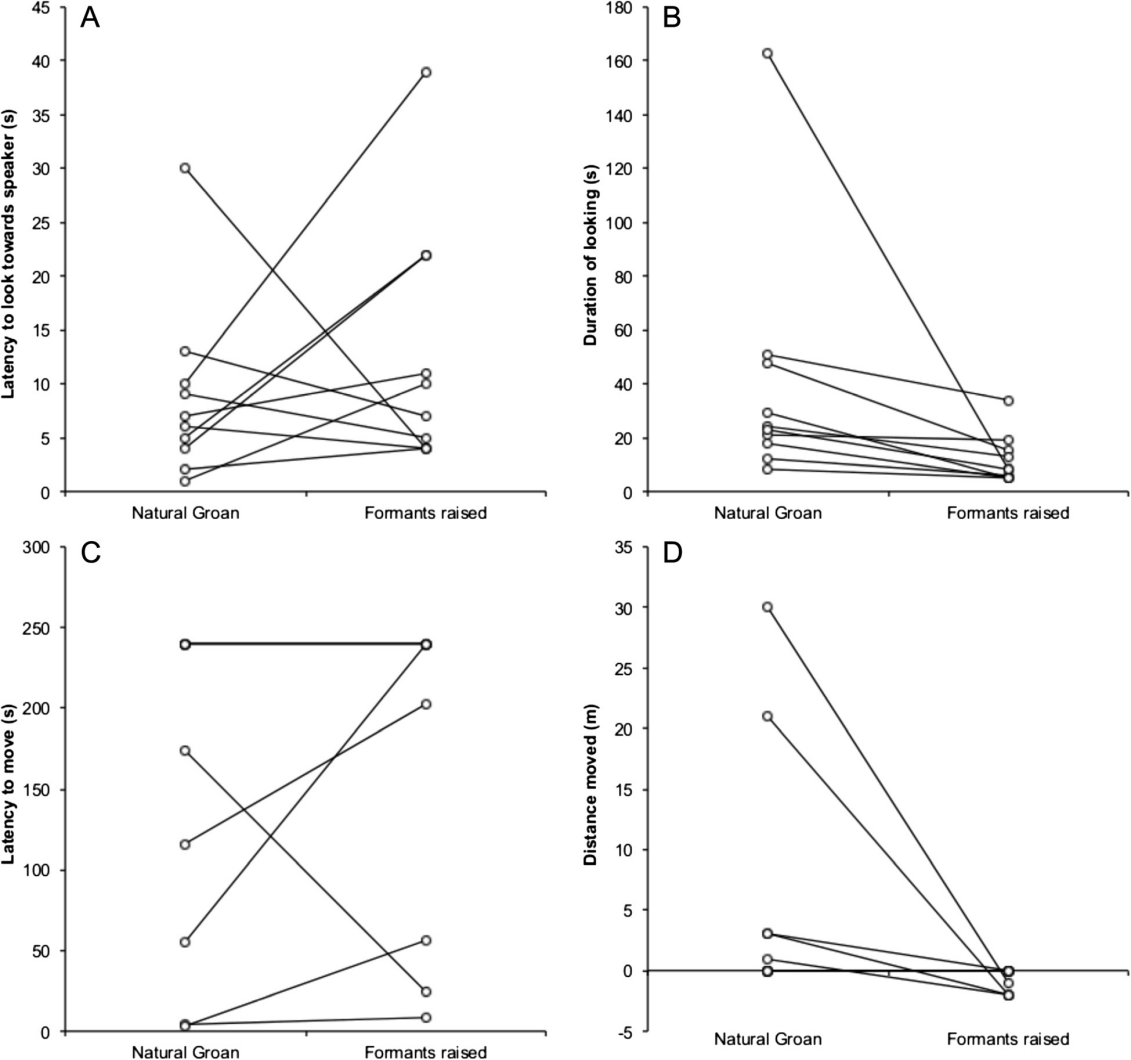
The responses of fallow bucks to natural groans and modified groans with raised formant frequencies. Pairs of points joined by lines represent the response of an individual buck. Bucks looked toward the speaker for less time (**b**) and were more likely to approach the speaker (**d**) in response to groans with raised formant frequencies than natural groans. There was no consistent difference in response in either the latency to look towards the speaker (**a**), or the latency to begin moving (**c**) between groan types (see Results for statistics)

Bucks responded to playbacks of groans with lowered formant frequencies by looking towards the speaker sooner than in response to natural groans (Fig. 2a. Wilcoxon signed-ranks test: Z = -2.25, N = 10, R = 0.71, P = 0.024). There was no difference in the duration of looking towards the speaker, or in the latency to move and the distance moved between lowered formants groans and natural groans (duration of looking: Fig. 2b. Wilcoxon signed-ranks test: Z = --1.79, N = 10, R = 0.57, P = 0.074; latency to move: Fig. 2c. Wilcoxon signed-ranks test: Z = -0.94, N = 10, R = 0.30, P = 0.345; distance moved: Fig. 2d. Wilcoxon signed-ranks test: Z = -0.31, N = 10, R = 0.10, P = 0.753). By contrast, bucks spent significantly less time looking towards the speaker (Fig. 3b. Wilcoxon signed-ranks test: Z = -2.80, N = 10, R = 0.89, P = 0.005), and were more likely to approach the speaker (Fig. 3d. Wilcoxon signed-ranks test: Z = -2.03, N = 10, R = 0.64, P = 0.042), in response to playbacks of groans with raised formants than to natural groans. There was no difference in latencies to look or move between natural and raised formant groans (latency to look: Fig. 3a. Wilcoxon signed-ranks test: Z = -0.87, N = 10, R = 0.28, P = 0.386; latency to move: Fig. 3c. Wilcoxon signed-ranks test: Z = -0.94, N = 10, R = 0.30, P = 0.345).

### Response to playback of groans with shortened duration

**Fig. 4:**
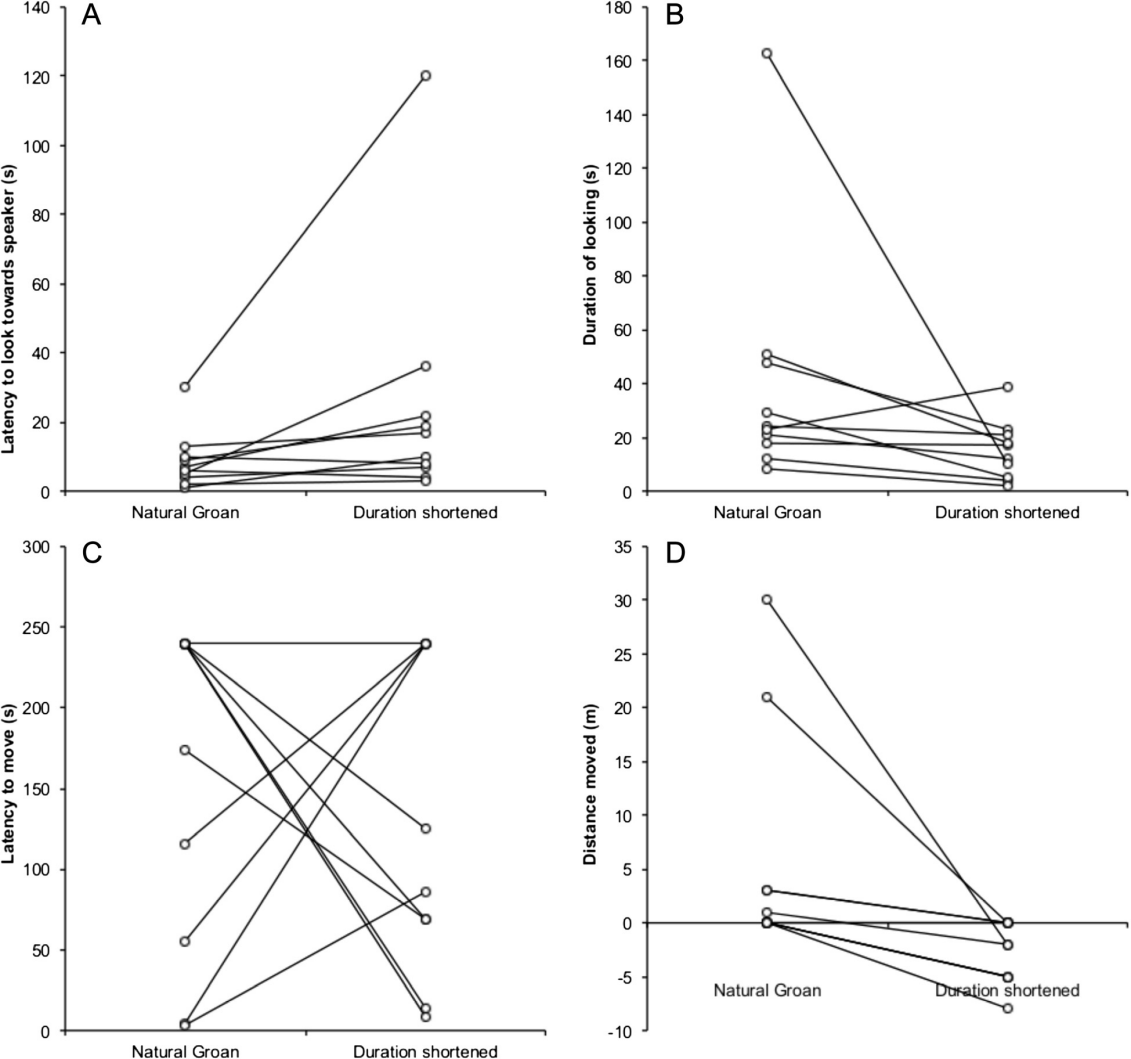
The responses of fallow bucks to natural groans and modified groans with shortened durations. Pairs of points joined by lines represent the response of an individual buck. Bucks were slower to look towards the speaker (**a**), looked for less time (**b**) and were more likely to approach the speaker (**b**) in response to groans with shortened durations than to natural groans. There was no consistent difference in response in the latency to begin moving (**c**) (see Results for statistics)

When presented with groans of shortened duration compared to natural groans, fallow bucks took longer to look towards the speaker (Fig. 4a. Wilcoxon signed-ranks test: Z = -2.30, N = 10, R = 0.73, P = 0.022) and looked for a shorter time (Fig. 4b. Wilcoxon signed-ranks test: Z = -2.19, N = 10, R = 0.69, P = 0.028). While there was no difference between the two stimuli in the latency to move (Fig. 4c. Wilcoxon signed-ranks test: Z = -0.30, N = 10, R = 0.09, P = 0.767), bucks were more likely to approach the speaker during playbacks of shortened duration groans than natural groans (Fig. 4d. Wilcoxon signed-ranks test: Z = -2.68, N = 10, R = 0.85, P = 0.007; Additional file 1).

In summary, fallow bucks looked more rapidly and for longer when the fundamental frequency or formant frequencies had been lowered, compared to natural groans. By contrast, bucks looked for less time and were more likely to approach the speaker during playbacks of groans with raised formant frequencies and shorter durations, when compared to natural groans.

## Discussion

Using playback presentations, we examined fallow buck perception of information about caller dominance, size and condition, contained in conspecific groans. We hypothesised that bucks would show greater attention and would not approach, or would retreat, when hearing groans indicative of more competitive callers, suggesting that they perceive these callers as more threatening. By contrast, we expected bucks to approach the speaker more when hearing groans indicative of less competitive callers. We presented bucks with groans that had been manipulated to alter vocal cues to these male characteristics compared to the same, non-modified (natural) groans. Our results show that bucks perceive changes in groan fundamental frequency, formant frequencies and duration. Bucks treated groans with lowered fundamental frequencies (simulating a more dominant caller) as more threatening than natural groans (i.e. faster reaction and longer duration of looking at the speaker). Groans with lowered formant frequencies (indicating a larger caller), were also treated as more threatening than natural groans (i.e. longer duration of looking at the speaker), while groans with raised formant frequencies (indicating a smaller caller) were treated as less threatening (i.e. shorter duration of looking and closer approach towards the speaker). Finally, groans with shorter durations (indicating a more fatigued caller) were treated as less threatening (i.e. slower reaction and shorter duration of look). We suggest that fallow bucks are able perceive information from these multiple parameters of conspecific vocalisations, in order to assess the level of competition posed by other bucks. It is likely that these functional responses of receivers have driven evolution at the level of vocal production to shape the vocal signals of fallow bucks [2, 40]. Furthermore, our results demonstrate the similarities between humans and fallow deer in the perception and use of both formants and *f*_*o*_ in vocal signalling [11, 41], highlighting potential common evolutionary pressures across mammals.

In fallow buck groans, *f*_*o*_ is negatively correlated with dominance rank [4, 5], and may provide cues to the hormonal state of callers [5]. We found that fallow bucks responded to groans with lowered *f*_*o*_ more rapidly and remained attentive for longer when compared to natural groans. Fallow bucks thus perceived information in the fundamental frequency of groans, and the behavioural response was consistent with *f*_*o*_ providing cues to the dominance rank of individuals; bucks were more attentive to groans with lower fundamental frequencies, which are typically associated with more dominant individuals. The very low *f*_*o*_ of fallow buck groans suggests that these vocal signals have evolved to broadcast information about the competitive ability of individuals [5, 34]. To our knowledge, this is the first experimental demonstration of the importance of *f*_*o*_ in male competition in a non-human mammal and contrasts with the findings for closely related deer species [42].

In stark contrast to fallow bucks, closely related red deer stags do not attend to *f*_*o*_ in conspecific roars [42]. Fallow buck vocalisations have a disproportionately lower *f*_*o*_ than female vocalisations [32], whereas in red deer, there is no sexual dimorphism in *f*_*o*_ and females prefer males with higher than average *f*_*o*_ [43]. In red deer, *f*_*o*_ typically decreases as males age [21], in contrast to fallow bucks, where *f*_*o*_ increases with age [4]. While the preference of female fallow deer for *f*_*o*_ has not been experimentally tested, more dominant males tend to have lower *f*_*o*_ and higher mating success [4, 5]. It is therefore likely that intersexual selection has driven the evolution of higher *f*_*o*_ in red deer stags, while intra- and potentially intersexual selection have led to the extremely low *f*_*o*_ of fallow buck groans.

The lower fundamental frequency of fallow buck groans may facilitate the assessment of body size through improving the resolution of formants [15, 44]. In humans, as in many mammals including fallow bucks, *f*_*o*_ and body size tend to be quite independent of one another within members of the same age and sex [2, 15], and *f*_*o*_ has been suggested to reduce the accuracy of voice-based size assessment, particularly when *f*_*o*_ information is discordant with formant information (e.g. a large individual with a high *f*_*o*_ or vice versa) [45]. However, Pisanski et al. [15] found that humans more accurately assessed speaker size when pitch cues were present, in contrast to when they were absent, e.g. whispered speech, despite the absence of a relationship between pitch and speaker height. Further, listeners performed better at assessing speaker size when pitch was lower and harmonics were denser than when pitch was higher and harmonics more sparse. Therefore it is likely that greater harmonic density generated by lower *f*_*o*_ allows better formant resolution and aids formant-based size assessment [15]. Indeed, ‘pulsatile’ vocalisations such as fallow deer groans and koala (*Phascolarctos cinereus*) bellows may further emphasise formant frequencies [25, 46]. In these vocalisations, glottal pulses contain broadband energy and are heard as individual events, such that pitch is not perceived [46]. Thus it is possible that very low *f*_*o*_ evolved in these species to increase the perception of formant-related information that indicates body size.

The *f*_*o*_ of groans may be selected to directly signal attributes such as hormonal levels. In human males, pitch is negatively correlated with testosterone levels [8–11], and low pitched voices are perceived as more dominant [47] and more masculine [10]. Testosterone causes immunosuppression, and thus is a good indicator of quality as only high quality males can express this costly trait [10, 48]. Among mammals, there is increasing evidence for salient indicators of hormone levels in vocal signals [49–51]. While further study on the effect of hormonal levels on vocal parameters is needed, it is possible that (like in humans) the low *f*_*o*_ of fallow buck vocalisations signals the hormonal levels of callers and thus their quality.

Formants correspond to resonance frequencies of the vocal tract and hence are strongly linked to its length [2]. The mobile descended larynx of fallow deer allows bucks to elongate their vocal tract by an average of 52 % during each groan, potentially giving receivers an exaggerated impression of the caller’s body size [33, 41]. Despite this mobile larynx, the minimum formant frequencies and minimum formant dispersion that an individual can produce when the larynx is fully retracted toward the sternum are an accurate cue to body size [5]. Larger males are typically higher ranked and have greater mating success, indicating that they are better competitors [29]. Formant frequencies may also provide information about the age of callers [4]. Prime-aged bucks aged 6 and 7 years old tend to produce groans with lower minimum formant dispersion, than when they are aged 5 or 8 years [4]. This change in formant dispersion probably reflects their maturation and subsequent senescence [4]. Our results demonstrate that fallow bucks are attentive to formant information in groans; males responded to groans with lower formant frequencies (i.e. simulating larger conspecifics) more rapidly than natural groans. By contrast, they responded to groans with higher formant frequencies (i.e. simulating smaller conspecifics) by looking for shorter periods, but were more likely to approach towards the speaker during playbacks. Thus, it seems that bucks perceived groans with lower formants as coming from larger, more threatening individuals, whereas groans with higher formant frequencies were perceived as originating from smaller, less threatening males.

The stability of vocal signals through the breeding season is likely to provide important information about the changing condition of callers [38]. Fallow bucks lose on average 26 % of their body weight during the rut [39], and as a result of declining body condition, groans become shorter and have higher *f*_*o*_ [38]. Fallow bucks can perceive these changes and respond less to fatigued groans than to groans from bucks in good condition [37]. Our results reveal that groan duration is one of the parameters that bucks are likely to be using to assess the condition of conspecific callers; bucks were slower to respond, were attentive for less time and were more likely to approach towards the speaker during a playback of groans with shorter duration (i.e. simulating a more fatigued conspecific), compared to a playback of natural groans. This suggests that fallow bucks detect changes in groan length and can use this to assess the state of callers, potentially gaining information about caller fatigue. Therefore, groan duration could be a product of intrasexual selection.

Despite being closely related [52], it appears that selection has acted differently on vocal production in fallow deer and red deer. Both species possess a mobile descended larynx that is retracted during vocalisations [41], and both use formant information when assessing conspecifics [53]. However, during roaring, red deer typically retract the larynx down to its maximum extent for each roar [21], whereas fallow bucks only retract the larynx by approximately 68 % of the distance between the snout and sternum during each groan [33]. Further, in contrast to fallow bucks, the roaring rate of red deer is much lower, typically a maximum of approximately 8 roars per minute [54], compared to approximately 54 groans per minute produced by fallow bucks during the rut [35]. Groaning rate is a very important signal for fallow bucks. Bucks modulate the rate of their vocal displays depending on the audience as a possible intrasexual threat signal [35, 36], and they perceive higher vocal rates as more threatening than lower rates [37]. Groaning rate and groan duration are inversely related, and because fallow bucks retract the larynx during each groan [33], the larynx cannot be retracted as far during shorter-duration but higher-rate groaning, for example during “harsh” groans [32, 55]. Our results have demonstrated that in fallow deer, the duration, *f*_*o*_ and formant frequencies, as well as groaning rate, provide salient information during intersexual competition and appear to be under strong sexual selection. By contrast, in red deer, although calling rate is an important cue [54], formant frequencies appear to be the dominant cue during intrasexual competition [21, 53], as stags do not attend to *f*_*o*_ [42].

While the results of this study are based on a limited sample size of ten individuals, they nevertheless provide important evidence for the selection pressures driving the evolution of the fallow buck vocal system. Fallow bucks display *f*_*o*_ that are far lower than predicted by their body size [32, 34] and modulate their formant frequencies during every groan by retracting the larynx [33]. Furthermore, the quality of a buck’s groan declines over the course of the rut [38], allowing competitors to assess the condition of other males [37]. The results support our hypothesis that lower *f*_*o*_ and formant frequencies are indicators of higher-quality bucks, while lower formant frequencies and shorter durations indicate lower-quality individuals. It is unlikely that bucks were simply responding to the novelty of the stimulus. Bucks responded as predicted to groan modifications, either treating modified groans as more or less threatening than natural calls. If bucks were simply responding to novelty we would expect the direction of responses to be similar for all novel stimuli.

## Conclusions

To conclude, this study demonstrates that, as a result of intrasexual competition, selection can act upon multiple parameters of vocalisations to simultaneously signal caller dominance, body size and condition. Further, to our knowledge, our results represent the first experimental demonstration of the importance of *f*_*o*_ in male competition in non-human mammals. Although to the human ear, the groans of fallow deer sound relatively simplistic and repetitive, we show that bucks have advanced perception abilities that allow them to extract information based on relatively small changes in some very salient parameters.

## Methods

We conducted this study in Petworth Park, West Sussex, UK (283 ha 50° 59’ 16” N, 0° 36’ 39” W), during the 2012 rut (late September to early November). The park has a population of approximately 700 fallow deer [37]. Some males form a lek during the breeding season [37, 56]. During the 2012 rut, at least 15 males were observed holding lek territories in the established lek area [56] and between 5 and 10 in a neighbouring, second lek area, 350 m to the north east. This study followed the *Association for the Study of Animal Behaviour Guidelines for the treatment of animals in behavioural research and teaching* [57]. Petworth Park is open to the public and the deer are habituated to the presence of people. The territorial behaviour and habituation to people allows for approaches close enough to conduct playback experiments [37, 56]. The animals were not handled during this study, and permission for the study was obtained from The National Trust.

We used a combination of pelage colour type and antler morphology to visually identify animals. Identification began in September 2012, prior to the rut, and a subset of the adult males from the population were identified using a combination of photos, sketches and notes of key features, such as obvious markings or antler formations. Only males later observed holding lek territories and vocalising were used in the playback experiments.

### Vocalisation recording

Recordings of bucks were made using a Sennheiser MKH 70 directional microphone in a Rycote Windshield and Windjammer, connected to a Marantz PMD 661 digital recorder. Vocalising males were recorded from between 10 and 40 m during daylight hours with a sampling rate of 44.1 kHz and amplitude resolution of 16 bits in WAV format. Recordings were made, between October 9 and 15 (inclusive), of adult males that had been observed holding lek territories.

### Acoustic analyses and groan resynthesis and construction

**Fig. 5:**
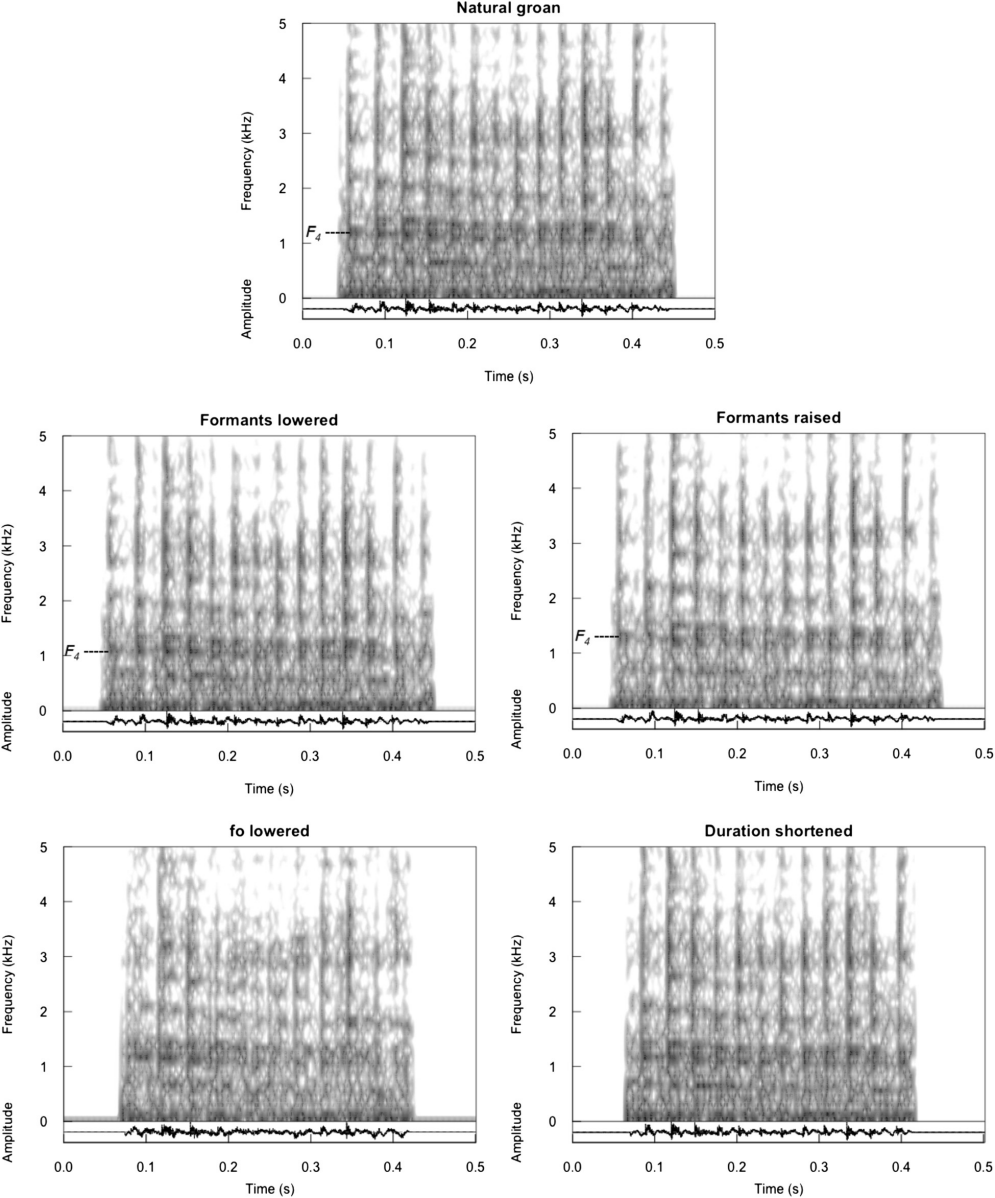
Spectrograms and oscillograms of a single groan and its modified variants from one individual. The natural groan was modified to produce the four variants. Five unique playback series were constructed for each of the ten experimental subjects. Playback series consisted of natural or modified groans repeated to represent a groaning bout. *F*_*4*_ indicates the fourth formant of the groan

Groans with good signal-to-noise ratios were extracted from recordings of 10 individuals (i.e. one for each subject tested with playbacks). From each groaning individual, a set of 5 playback stimuli was prepared (Fig. 5, Additional files 2, 3, 4, 5 and 6) consisting of the following: 1) natural groans (no manipulation); 2) same groans with fundamental frequency shifted down; 3) formant frequencies shifted up; 4) formant frequencies shifted down; 5) and shortened duration. Because of the manipulation method used, it was not possible to generate a stimulus with raised fundamental frequency.

Manipulation of groans was conducted using PRAAT [58] and Adobe Audition 3 (Adobe Systems Incorporated, San Jose, CA). We constructed the signals used in the playback experiment by resynthesising the natural groans, modifying either their formant frequencies or their fundamental frequency. Further, to create groans of shortened duration, recordings of natural calls were trimmed to the required duration.

The fundamental frequency, formant frequencies, and duration for each natural groan were measured in PRAAT [58] following the procedure described in Briefer et al [4]. The measurements extracted from each groan were used to determine the suitable modification factors by which to modify the groans. Analysis of 305 groans recorded from 14 males from the population at Phoenix Park, Ireland (707 ha, 53° 21’ N, 6° 19’ W) by Briefer et al [4] was used to determine population means (Table 1). The groans of deer from Phoenix Park are very similar to those from Petworth Park [59]. Modification factors of 1.5 times the standard deviation of the population means were selected to ensure that the modified vocalisations were different to the natural vocalisation, but remained within the natural range of the species (Table 1). This resulted in the following modification targets: *f*_*o*_ shifts of 6 Hz down, formant frequency shifts of 68 Hz up and down, and duration changes of 12.6 % shorter. The mean modifications to groans used in the playback experiment are shown in Table 2.

**Table 1:** Population means of vocalisation parameters (Briefer et al. [4])

Parameter	Mean	Standard Deviation	Range
*f*_*o*__mean_ (Hz)	28.3	3.97	18.9 – 42.7
*F*_*4*__min_^1^ (Hz)	1057.8	45.05	942.5 – 1221.4
Duration (ms)	385.3	8.8	214-758

^1^ *F*_*4*__min_ was used as an exemplar for all formants

**Table 2:** Mean modifications of groans used in the playback experiment (mean ± s.d)

Parameter	Natural	Raised formants	Lowered formants	Lowered *f*_*o*_	Shortened duration
*f*_*o*__mean_ (Hz)	29.3 ± 2.4	uc	uc	23.4 ± 2.4	uc
*F*_*4*__min_ (Hz)	1049.3 ± 67.6	1118.0 ± 53.5	1000.8 ± 65.6	uc	uc
VTL (cm)^1^	52.9 ± 3.3	49.0 ± 3.3	57.3 ± 3.9	uc	uc
Duration (ms)	370 ± 66	uc	uc	uc	320 ± 57

^1^ The apparent maximum vocal tract length (VTL) was estimated using the equation $VTL=\frac{c}{2\varDelta F}$ where c = the approximate speed of sound in a mammal vocal tract (350m/s) and Δ*F* is the minimum formant spacing [21, 62, 63]

*uc* unchanged parameter

### Modification of groan fundamental frequency

Because of the extremely low fundamental frequency of fallow buck groans [5], it was necessary to modify the fundamental frequency by removing the spectral variation in the signal, leaving only the pulse information. This signal was then stretched to achieve the desired pulse rate and resampled back to the original sampling frequency. Finally, the signal was trimmed to the duration of the original file, in order to keep the duration unchanged, before being filtered to reinstate the original formant information. To sample the spectral information in the natural signal, the command “To LPC (burg)” was used. The original signal was then filtered with the LPC file using the command “Filter (inverse)” to remove all spectral variation, leaving only pulse information. The new sound file was inspected visually to ensure that all formant information had been removed during the filtering. The duration of the sound file was lengthened by a predetermined resynthesis factor using the command “Scale times by” and then resampled to the sampling frequency of the original sound file using “Resample”. This resulted in a signal with the desired fundamental frequency. The sound file was then edited so that the duration was the same as the original, unmodified signal. Finally the signal was filtered with the original LPC file using the command “Filter” to reinstate the original spectral information. The final sound file was inspected to ensure that the desired modification had occurred and that no artefacts were introduced. Because of the method used to modify the fundamental frequency, it was not possible to produce a modified groan with an increased fundamental frequency without also shortening the duration of the groan. For this reason a playback using increased fundamental frequency was not included.

### Modification of groan formant frequencies

A custom PRAAT script was used to modify the formant frequencies of groans using a using a PSOLA (Pitch Synchronous Overlap and Add) based algorithm. The manipulation process shifted the formant frequencies up or down by a predetermined resynthesis factor, leaving the other acoustic parameters (e.g. *f*_*o*_) unchanged. Following resynthesis, the formant frequencies of the new signal were measured and the sound examined to ensure that the desired modification had occurred and no artefacts were introduced.

### Modification of groan duration

Modifications to the duration of groans were achieved by trimming the original sound file to the desired duration. Because fallow buck groans are characterised by a down sweep in formant frequencies [33], we removed equal durations from the beginning and end of the natural groan to modify both the higher and lower frequency segments of the groan.

### Playback construction

Using the natural and resynthesised groans described above, five playback series were constructed for each of the 10 recorded individuals. These consisted of a natural groan series, a series with raised formants, a series with lowered formants, a series with lowered fundamental frequency and a series with shortened duration. The natural or modified groan was repeated to represent a groaning bout of 1 min duration at a rate of approximately 44 groans per minute, simulating a high, but submaximal threat [35, 37]. Sequences were normalized to 95 % and saved as 44.1 kHz, 16 bit.wav format sound files for playback using Adobe Audition 3 (Adobe Systems Incorporated, San Jose, CA).

### Playback procedure

Playbacks were performed between 15 and 27 October around the estimated peak of mating activity (20–30 October: [56, 60]). Playbacks were presented to mature male subjects that were observed holding lek territories using a similar method to Pitcher et al [37]. The playbacks started when male subjects were not in close proximity (<100 m) to the male used to construct the playback, and had not been observed interacting with the male used in the playback in the previous 48 h. Groans were broadcast at an approximately natural amplitude (51 dBA at 15 m [37]) and were played from a Mackie Thump TH-12A loudspeaker (LOUD Technologies Inc., Woodinville, WA) connected to an Edirol R-09 (Roland Corporation, Los Angeles, CA) via a 40 m lead. The speaker was located at 43 ± 3.5 m (mean ± s.d.) from the subject and obscured using camouflage netting. Playbacks were conducted during calm weather and were not attempted on days with heavy rain and/or strong wind. The experimenter controlled and filmed the playbacks from 20-30 m behind the speaker, where possible obscured from the view of the subject. The speaker was placed at the appropriate location, a minimum of 5 min prior to the playback. Playback order was pseudo-randomised between individuals and a minimum of 30 min elapsed between playback stimuli, allowing individuals to resume normal behaviour. Each male was presented with all variants of the playback treatments. For 3 of the 10 subjects it was not possible to present all playbacks within one day. Playbacks to two of the subjects were completed on the following day, while playbacks to the third were completed after two days. The distance between the subject and the speaker was measured at the beginning and end of the presentations using a Tasco 400 rangefinder. A presentation period consisted of 60 s of playback followed by an additional 180 s of observation and filming with a Canon LEGRIA camera.

### Data analysis

Following a playback presentation, the experimenter measured the distance moved towards or away from the speaker by the subject during the presentation period, relative to its starting location. Other responses of subjects to the playbacks were measured from the video recordings after the experiment. The video observer measured the latency from the beginning of the playback presentation until the subject looked towards the speaker, and the duration of looking during the presentation period (240 s in total; i.e. 60 s of playback and 180 s of post-playback observation). The observer also measured the time taken for the subject to start moving after the beginning of the playback. These responses were used to assess whether bucks perceived the stimuli as more or less threatening than a natural groan. Fallow bucks engage in agnostic behaviour by approaching competitors to parallel walk or fight [61]. We therefore interpreted bucks moving toward the source of the groans as willing to engage in agonistic behaviour whereas those that retreated were moving away from a potential threat. Similarly, we expected bucks to be more attentive, and therefore look towards the source of the groans faster and for longer, in more threating situations. This measure of threat has been used in previous studies of both fallow deer and red deer [37, 53]. Wilcoxon signed-ranks tests were used to compare the responses of subjects to the various modified stimuli with their response to the natural groans. Statistical analyses were conducted using SPSS 16.0 for Windows (SPSS Inc, Chicago, IL, USA).

## Availability of supporting data

The data set supporting the results of this article is included within the article and its additional files. See Additional file 7.

## Additional files

Additional file 1:
**Playback video.** This video shows the playback of groans with a shortened duration to a fallow buck at Petworth Park on 26 October 2012. (MP4 19276 kb) Additional file 2:
**Natural groan.** An example of a single natural groan. (ZIP 34 kb) Additional file 3:
**fo lowered.** An example of a single groan that has been modified to lower the fundamental frequency. (ZIP 31 kb) Additional file 4:
**Formants lowered.** An example of a single groan that has been modified to lower the formant frequencies. (ZIP 34 kb) Additional file 5:
**Formants raised.** An example of a single groan that has been modified to raise the formant frequencies. (ZIP 34 kb) Additional file 6:
**Duration shortened.** An example of a single groan that has been modified to shorten the duration. (ZIP 31 kb) Additional file 7:
**Pitcher et al data.** Dataset of responses of fallow bucks to playback of natural and modified groans. (XLSX 48 kb)

## Abbreviations

*f*_*o*_: Fundamental frequency
ha: Hectare
Hz: Hertz
kHz: Kilohertz
m: Metres
min: Minutes
PSOLA: Pitch Synchronous Overlap and Add
s: Seconds
